# Brain mapping and detection of functional patterns in fMRI using wavelet transform; application in detection of dyslexia

**DOI:** 10.1186/1472-6947-9-S1-S6

**Published:** 2009-11-03

**Authors:** Soo-Yeon Ji, Kevin Ward, Kayvan Najarian

**Affiliations:** 1 Department of Computer Science, Virginia Commonwealth University, 401 East Main Street, Richmond, Virginia, USA; 2 Department of Emergency Medicine, Virginia Commonwealth University, 1201 E. Marshall St, Richmond, Virginia, USA

## Abstract

**Background:**

Functional Magnetic Resonance Imaging (fMRI) has been proven to be useful for studying brain functions. However, due to the existence of noise and distortion, mapping between the fMRI signal and the actual neural activity is difficult. Because of the difficulty, differential pattern analysis of fMRI brain images for healthy and diseased cases is regarded as an important research topic. From fMRI scans, increased blood ows can be identified as activated brain regions. Also, based on the multi-sliced images of the volume data, fMRI provides the functional information for detecting and analyzing different parts of the brain.

**Methods:**

In this paper, the capability of a hierarchical method that performed an optimization algorithm based on modified maximum model (MCM) in our previous study is evaluated. The optimization algorithm is designed by adopting modified maximum correlation model (MCM) to detect active regions that contain significant responses. Specifically, in the study, the optimization algorithm is examined based on two groups of datasets, dyslexia and healthy subjects to verify the ability of the algorithm that enhances the quality of signal activities in the interested regions of the brain. After verifying the algorithm, discrete wavelet transform (DWT) is applied to identify the difference between healthy and dyslexia subjects.

**Results:**

We successfully showed that our optimization algorithm improves the fMRI signal activity for both healthy and dyslexia subjects. In addition, we found that DWT based features can identify the difference between healthy and dyslexia subjects.

**Conclusion:**

The results of this study provide insights of associations of functional abnormalities in dyslexic subjects that may be helpful for neurobiological identification from healthy subject.

## Background

Experimental approaches towards the study of the brain functions often involve the detection of brain regions that respond differently to external stimuli. Thus, several researchers [1,2] studied the brain activity when specific external stimuli is incited. An effective technology to study the human brain is functional magnetic resonance imaging (fMRI). It measures blood oxygenation-level-dependent (BOLD) signal changes caused by hemodynamic response of neural activity [3,4]. However, due to the presence of different sources of noise and distortion, the mapping between the fMRI signal and the actual neural activity requires significant filtering and signal processing [5]. For instance, unwanted changes in fMRI signal intensity can be caused by head motions as a main source of noise and distortion; this is commonly referred to motion artifact [6]. Because of these difficulties, differential analysis of healthy and diseased cases using signal processing and pattern analysis of fMRI brain images remains as one of the main research challenges in the field [7].

Functional MRI is a well known technique for studying physiological conditions of the brains in human subjects. It is a non-invasive technology that tracks the changes in BOLD responses related with neural activities. From fMRI scans, the increased blood flow to the activated areas of brain is detected [8]. Based on a series of multi-slice images of the brain, fMRI provides the functional information of detecting and analyzing different parts of the brain [9,10]. Through the analysis of dynamic views of brain activities, the functions and high-level cognitive tasks can be studied. For instance, Ohnishi [11] shows that the classification of morphologic changes in the brain with normal aging and Alzheimer disease is different [12]. Gallaghera [13] provides functional imaging studies on 'theory of mind' in verbal and non-verbal to identify distinct active region. Since fMRI signals are generated by the changes of BOLD in neural activity, the functional role of neural activity might be addressed by detecting the activated areas [14].

Dyslexia is a significant disease of reading acquisition [15]. Habib [16] indicates that in spite of appropriate educational opportunities, five to ten percent of school students do not have reading ability simply because they suffer from dyslexia. Dyslexia, which is defined as a specific disability of reading and learning, was first identified about 100 years ago as a learning imparity, while at the early stage dyslexia is often taken as a vision problem [17,18]. Nowadays, fMRI can determine that when people with dyslexic patients read, they do use different parts of their brain compared to people without dyslexia [19]. Many previous studies [20-22] discuss investigations of the specific brain activities regions involved in dyslexia. According to the studies [1,23], posterior part of the left superior temporal gyrus and the inferior parietal gyrus (BA (Broadmann area) 40) are the most likely to involve language processes. However, the most significant difficulty in dyslexia is the lack of identifying the patterns of dyslexia.

Therefore, a possible approach to identify dyslexia and healthy subjects is to adopt a signal processing technique to extract valuable information from the fMRI signal of interested regions. In our previous study [24], we proposed a signal processing technique using an optimization theory, called modified MCM, to filter, process, and classify the fMRI images. To analyze the fMRI time-series, pixel-regions in fMRI are used to explore the brain activities that significantly improves conventionally maximum correlation modeling (MCM) of the brain hemodynamic response across the healthy and dyslexia subjects. The intention of this paper thus as continuation of our previous work is that presenting the improvements of filtering technique developed in our previous study to compare the brain activities of the healthy and dyslexic subjects. In addition, the utility of discrete wavelet transform (DWT) to identify different patterns between healthy and dyslexia subjects is presented. Simple statistics based on mean and standard deviation quantitative analysis is used to provide knowledge of the signals' significance. However, standard deviation and mean may not provide an appropriate characterization of the rapid changes in a signal. For this reason, further measurement, Higuchi fractal dimension (FD) and discrete wavelet transform (DWT) features are used to describe the difference between before and after signal filtering applied. Before filtering indicates a raw time series of single activated pixel at regions of interest based on statistical parametric mapping (SPM) and filtering signal suggests that the results of linearly combined signal with neighbors' pixel of the activated single pixel using a practical optimization algorithm. FD analysis is a useful tool in the identification of complexity under different conditions. In other words, FD is useful for measuring self-similarity of the signals. For this study, Higuchi FD is used because it is easy and simple to implement [25]. Wavelet transform analysis [26,27] is a very promising signal processing technique to detect abnormal changes within the signals. In particular, DWT decomposes the signals at different scales and resolutions. Thus, it is suitable for analyzing non-stationary signals.

Our hypotheses for this study are as follows:

1. We hypothesize that signal filtering using a hierarchical optimization algorithm with MCM developed in our previous study helps to enhance the signal activity of interested regions.

2. We hypothesize that the features extracted from the signals using wavelet transformation may help to differentiate between healthy and dyslexia subjects.

**Figure 1:**
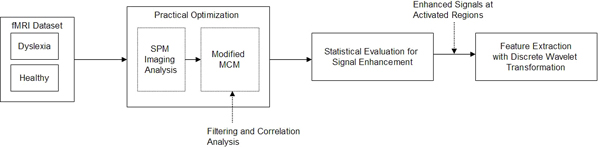
**Schematic diagram of proposed method**. This figure presents a detailed schematic diagram for the our proposed method.

Figure 1 presents a schematic diagram of the proposed processing methods. As mention above, two groups of fMRI images, i.e. for dyslexic and healthy subjects, are collected. First, SPM is applied to identify the most significant regions across the two groups. A modified MCM, in which correlation of the parameters are quantitatively analyzed, is applied to process the selected regions. Then, wavelet transformation signal processing technique is applied to the filtered signals in order to reveal the informative patterns between healthy and dyslexic subjects after performing the statistical evaluation of the filtered signals enhancement. The results are compared.

## Methods

### Description of dataset

**Figure 2:**
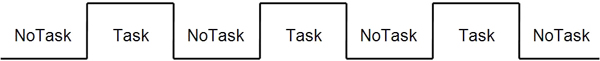
**Boxcar diagram procedures**. This figure shows the stimuli protocol used in the study ("NoTask" indicates not showed words and "Task" represents showed words).

The fMRI data for both healthy and dyslexic subjects are collected using a 1.5-T General Electric echo-speed Horizon LX scanner with a birdcage head coil (GE Medical Systems, Milwaukee, WI) by Radiology Department of Wake Forest University. Ten fMRI scans are used for this study of which half are from healthy subjects and the rest belong to dyslexic patients. Data are collected with a word recognition stimulus, in which sixteen words are projected onto the screen for 32 seconds. A new set of words are shown to each subject every 2 seconds. No visual task is a state when the screen is blank. For the visual and no-visual tasks, a block design paradigm, called boxcar, is used to represent blocks' functionality. The boxcar contains five blocks of active and rest periods with 32 seconds of visual task and no-visual task, respectively. Figure 2 represents boxcar diagram procedure of repeating two behaviors (i.e. task and notask) for this study.

Each functional scan consists of 96 sequential images as an echo-planar sequence. Parameters for these scans are: TR = 2500 *ms*, TE = 50 *ms*, and flip angle is set to 90. Each image consists of 28 transaxial slices that are 5 *mm *thick (no space between slices) with a field of view of 24 cm (frequency) × 15 *cm *(phase) and an acquisition matrix of 64 × 40 resulting in an in-plane resolution of 3.75 *mm*. High-resolution anatomic images are collected using an inversion recovery 3*D *spoiled gradient echo sequence with the following parameters: matrix is 256 × 256, field of view is 24 *cm*, section thicknesses 3 *mm *with no gap between slices, number of sections is 60, and in-plane resolution is 0.94 *mm*. This sequence is used both in anatomic overlays of the functional data and in spatial normalization of the data sets with a standard atlas.

### Practical optimization

The detail description of the practical optimization is introduced in our previous study [24]. In briefly describing the method, the practical optimization algorithm contains two part i.e. SPM imaging analysis and modified MCM. The identification of specifically informative regions is considered in terms of dyslexic and healthy subjects with external stimuli (i.e. showing words (Task) and not showing words (No task)). Figure 2 depicted this boxcar diagram processes. In order to detect the highly informative regions, SPM is used [6,28,29]. For the interesting region selection, the statistical threshold is set to *p <*0.05, uncorrelated to find the most activated regions. For this study, six interested pixels which is activated when extrenal stimuli is applied between two groups are selected and used for further analysis.

Then, a significantly improved and modified version of Maximum Correlation Modeling (MCM) method presented by Friman [10] to detect interesting brain region's activities is described. MCM method is based on time series modeling of pixel neighborhoods (3 × 3) for detecting active parts that contain a significant hemodynamic response. To use the method, first we consider the regions in fMRI images and the time series of the pixels in the region. In order to estimate the activity of center pixel in each region, the activities of the symmetric neighbors of the center point are linearly combined using a vector weight which is determined by maximum correlation between the signals (time series) and known hemodynamic wave pattern. In order to maximize the correlation coefficient between the parameters of the time series and the hemodynamic response, an optimization process was conducted. This optimization process finds the best set of parameters under given constraints that identifies the best match between the expected hemodynamic response and the combined time-series. A more detail description of this algorithm is explained in our previous paper [24].

### Statistical evaluation for signal enhancement

We evaluate the performance of filtering of our proposed method introduced in previous study. In our previous study, we showed a different pattern of pixels activity and its correlation between healthy and dyslexic subjects. Now, the performance of signal improvement with the proposed method is evaluated. Before filtering (time series is presented using only a single pixel which is activated) and after filtering (time series is presented with linear combination of neighbor's pixels) signals are therefore compared with statistical analysis software (SAS).

As mention above, FD and DWT features are used for this comparison. FD [25,30] is the complexity measurement of objects, which are repeating the same patterns. Fractals have the characteristic that each subset is similar to the whole set, and FD is a measure of this self-similarity. The Higuchi FD, as explained below, is applied for this study. This method first re-generates the original signal as a finite time-series based on pre-defined fragment size. In our study, 8 and 15 fragment size are applied and its results are compared. For a given input signal *x*(1), *x*(2),⋯, *x*(*N*), the new finite time-series, , is constructed as follows:

(1)

where " []" denotes the floor function, that is, the greatest integer that is less than or equal to the value, and both *k *and *m *are integers representing the initial time and interval. Then the length of the curve *L*_*m*_(*k*) is defined as follows:

(2)

where  represents the normalization factor for the curve length and *N *is the total length of the signal. *< L*(*k*) *> *is defined as the length of the curve for the time series *k *and *< L*_*m*_(*k*) denotes the average value over *k*. Thus, if *< L*(*k*) > ∞*k*^-*D*^, then the curve has dimension *D*. In other words, FD identifies the slope of the best fit-line at the *log*-*log *plot for *log*(*L*(*k*) *<*versus *log*(*k*) [25].

### Feature extraction with discrete wavelet transformation

DWT is suitable for detecting and analyzing the changes of signal. Since wavelet transformation provides desirable characteristics in time-frequency signal processing [26,27], it is suitable for analyzing the time-varying characteristics of non-stationary signals. As mentioned earlier, DWT provides time information that is obtained by decomposing the signal into its frequency subbands with a series of lowpass and highpass filters. The output of the highpass filters at level *i *is the detail coefficient, which explains the fast changes of signals, whereas the output of lowpass filters is approximation coefficient. In this study, level 3 with Daubechies wavelet (db4) is performed. The features are written as follows:

(1) Sum of coefficients at each level, i.e.  where *d*_*i *_is detail coefficient of each level at *i *and *n *is a total length of coefficients.

(2) Variance of coefficients at each level, i.e. , where *μ *is a mean of each coefficient at level *i*.

(3) Median absolute deviation (MAD) of coefficients at each level *i*, i.e. *mad*(*i*) = *median*|(*d*_*i *_- *median*(*d*_*i*_))|, where *d*_*i *_is detail ceofficient at level *i*.

## Results

In this section, first the results between optimal filtering with neighbors' pixels and raw time-series activated single pixel on fMRI data of five healthy and five dyslexic subjects are presented as described in Method section. Then, the results of DWT to differentiate between healthy and dyslexia with ANOVA are presented.

First, sign rank sum test is performed to compare the optimal filtering improvements among within subjects (Table 1). For this comparison, Higuchi FD and DWT are used. As shown in Table 1, filtering based on practical optimization algorithm showed a significant difference within healthy and dyslexia subjects using all features including FD and DWT. This fact indicates that the filtering with practical optimization algorithm helps to enhance the signal activity.

**Table 1:** The comparison between optimal filtering and before filtering using FD and wavelet based features among within subjects (*d*_*i *_indicates the detail coefficient at level *i*).

	**Within Healthy Subjects**	**Within Dyslexia Subjects**
**Feature**	**p value**	**p value**

Sum of d_1_	0.0039	*<*0.0001
Variance of d_1_	0.0039	*<*0.0001
Mean absolute deviation of d_1_	0.0273	*<*0.0001
Sum of d_2_	0.0195	*<*0.0001
Variance of d_2_	0.0195	*<*0.0001
Mean absolute deviation of d_2_	0.0391	*<*0.0001
Sum of d_3_	0.0195	*<*0.0001
Mean absolute deviation of d_3_	0.0391	*<*0.0001
Higuchi FD (win = 8)	0.0078	0.0391
Higuchi FD (win = 15)	0.0039	0.0039

**Figure 3:**
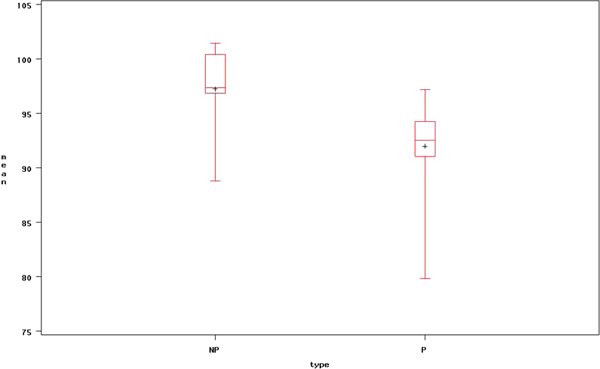
**Box plot of overall mean with filtering between healthy and dyslexic subjects**. This figure presents the average time-series signal after optimal filtering applied to healthy and dyslexic subjects (NP indicates an healthy case and P indicates dyslexia case).

**Figure 4:**
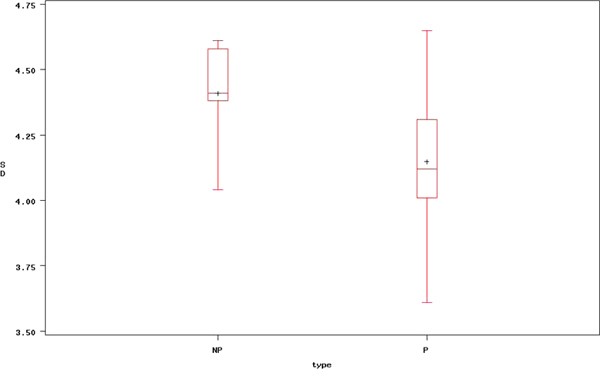
**Box plot of overall standard deviation between healthy and dyslexia subject**. This figure presents the average time series signal after optimal filtering of dyslexia subject (NP indicates an healthy case and P indicates dyslexia case).

Figure 3 and 4 depict boxplots for mean and standard deviation (SD) of healthy and dyslexia subjects, respectively. The mean of dyslexia subjects is lower than healthy subjects and variability of healthy subject is much wider than dyslexia subjects.

**Figure 5:**
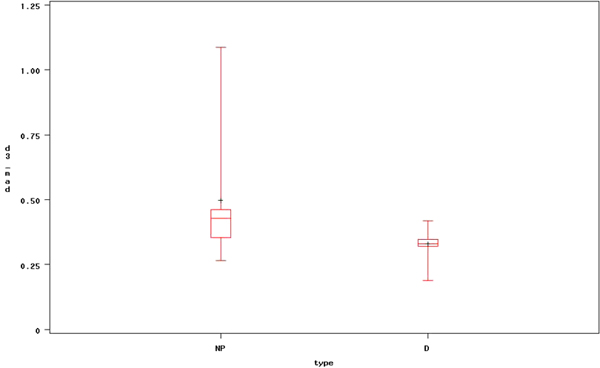
**Box plot of mean absolute deviation at level 3 (d_3_) of heanthy and dyslexia subjects**. This figure presents the box plot, which is one of the significant wavelet features, that can differentiate healthy subject from the dyslexia subjects (NP and P indicate healthy and dyslexia, respectively).

Next, ANOVA analysis is performed with the same features in Table 1 to evaluate the capability of differentiating between healthy and dyslexia subjects. According to the ANOVA test, we found that mean absolute deviation of detail coefficients at level 2 (*p *= 0.0457) and level 3 (*p *= 0.0273) with wavelet transform is significant which means that they can distinguish between healthy and dyslexic subjects. Figure 5 depicts a box plot of the mean absolute deviation at level 3 features between healthy and dyslexic subjects.

## Discussion

In our previous study, we presented the brain activation using a hierarchical optimization algorithm. In particular, an optimization algorithm is applied to specific regions of the brain identified by SPM to find patterns that are significantly different across two groups of subjects.

In our study, fMRI analysis is performed based on the activated areas for the tasks (showing the words/not showing the words). It must be addressed that we do not emphasizes the issue of the statistical significance of activations that are detected in this paper. Our approach allows us to compare the improvements of filtering and provides the capability of identification between healthy and dyslexic subjects with wavelet transform method.

The main aim of this paper is to not only compare the performance between before and after filtering using practical optimization algorithm with fractal dimension and DWT features, but also evaluate the capability of wavelet based features to distinguish between two groups. DWT and FD can show the improvements of signal filtering by comparing with single pixel time-series of brain activation. However, fractal dimension does not show that they can distinguish the different conditions. This finding demonstrates that the practical optimization algorithm can help to improve the filtering to obtain the brain activation signals. In addition, we found that wavelet based features may be useful to distinguish the healthy and dyslexic subjects. This fact supports the opportunity of using wavelet analysis to distinct different condition. Thus, it may possible to use early decision strategy to identify the abnormal activation of the brain in fMRI data. The key point of applying the wavelet transform, which is a multi-resolution method is selecting of the appropriate wavelet bases functions. Since there is no ultimate way of choosing the wavelet, one of Daubechies families, db4, is used for this study. But, the question still remains, what is the best choice of wavelet to extract the valuable information from the signal. Also, the detail coefficients is contained data variation information at a particular subband. Thus, they can present a knowledge which is contained within the dataset. For this study, detail coefficients at level 2 and 3 are only significant to discover two subjects. The detail coefficient at level 1 which is associated with the most high frequency of data may not provide big difference between two subjects due to the pattern similarity.

A limitation of this study is the small size of the dataset used to identify the fMRI brain activity. However, despite the small size of the dataset, the method based on DWT features successfully differentiates the two groups, healthy and dyslexia subjects. Therefore, additional analysis with a large amount of dataset with many pixels should be performed to test between dyslexic and healthy cases.

## Conclusion & future work

As a continuation of our previous work, the purpose of this work is not only to evaluate the improvement of filtering with the hierarchical optimization algorithm, but also apply a signal processing strategy to identify two subjects, healthy and dyslexia subjects.

In this paper, we present the utility of practical optimization algorithm based on FD and DWT features. In addition, we describe that wavelet based features may useful to differentiate two groups, healthy and dyslexic subjects, of fMRI time series. In this study, we perform wavelet transform as an appropriate non-stationary signal analysis method, which may be suitable for differentiating two different conditions. For future work, more datasets with many regions will be tested and used to evaluate the two groups, non-dyslexic and dyslexic subjects. Also, other possible wavelet-based features are going to be identified for differentiating them.

## Competing interests

The authors declare that they have no competing interests.

## Authors' contributions

All authors have equal participation in the study as well as preparation of the final paper.
